# Preoperative 7 T 3D Meniscal T2* Mapping Correlates With Postoperative Extrusion After Medial Meniscus Posterior Root Tear Repair

**DOI:** 10.1002/jor.70286

**Published:** 2026-10-07

**Authors:** Abdul Wahed Kajabi, Karsten Knutsen, Eisa Hedayati, Collin Steinberger, Štefan Zbýň, Asif Abul Hassan, Hassan Ahad, Abhinav Lamba, Luke V. Tollefson, Takashi Takahashi, Gregor J. Metzger, Robert F. LaPrade, Jutta M. Ellermann

**Affiliations:** ^1^ Center for Magnetic Resonance Research University of Minnesota Minneapolis Minnesota USA; ^2^ Department of Radiology University of Minnesota Minneapolis Minnesota USA; ^3^ Department of Biomedical Engineering Cleveland Clinic Research, Cleveland Clinic Cleveland Ohio USA; ^4^ Twin Cities Orthopedics Edina Minnesota USA

**Keywords:** 3D T2* mapping, collagen fiber organization, imaging biomarkers, meniscal extrusion, meniscus, quantitative MRI, root repair, root tear, ultrahigh Field 7 T

## Abstract

Medial meniscus posterior root tear (MMPRT) repairs stabilize root attachment and yield improved clinical outcomes, yet many patients develop persistent meniscal extrusion and osteoarthritis. This study evaluates menisci in MMPRT patients using 3D T2* mapping and assesses T2* correlation with medial meniscal extrusion. Patients underwent 7 T knee MRI before root tear repair and again at 6‐month postoperative follow‐up. Age‐ and gender‐matched controls were scanned once. The protocol included clinical turbo‐spin‐echo sequences and a quantitative 3D GRE sequence, used to calculate T2* mapping of the menisci. Pre‐ and postrepair meniscal extrusion was measured on coronal T2‐weighted images by two independent radiologists. Twenty‐five patients (mean age 53 ± 8 years) and nine controls (51 ± 11 years) were included. Repairs were intact on follow‐up MRI in all patients. T2* values were significantly elevated in patients both pre‐ and postrepair compared with controls across medial and lateral meniscal subregions. Medial meniscal extrusion increased from 3.7 ± 1.1 mm at baseline to 4.9 ± 1.5 mm (*p* = 0.002), postoperatively. Prerepair meniscal T2* strongly correlated with postrepair extrusion (*ρ* = 0.765; *p* < 0.001). High‐resolution 3D T2* mapping at 7 T revealed persistently elevated T2* values after MMPRT repair, consistent with ongoing collagen fiber disorganization. Prerepair T2* values were strongly correlated with early postrepair meniscal extrusion despite MRI‐intact repairs, indicating that preoperative tissue quality is associated with postoperative outcomes. These findings support quantitative T2* mapping as a prognostic imaging biomarker that complements conventional morphologic MRI in the evaluation of MMPRTs.

## Graphical abstract

Patients underwent 7 T knee MRI with routine clinical imaging and 3D T2* mapping before and 6 months after medial meniscus posterior root tear (MMPRT) repair. High‐resolution T2* mapping revealed persistently elevated T2* values, consistent with ongoing collagen fiber disorganization, despite MRI‐intact repairs. Elevated preoperative T2* values were strongly associated with postoperative meniscal extrusion. Quantitative T2* mapping provides a prognostic imaging biomarker that complements conventional morphologic MRI in the evaluation of MMPRTs.

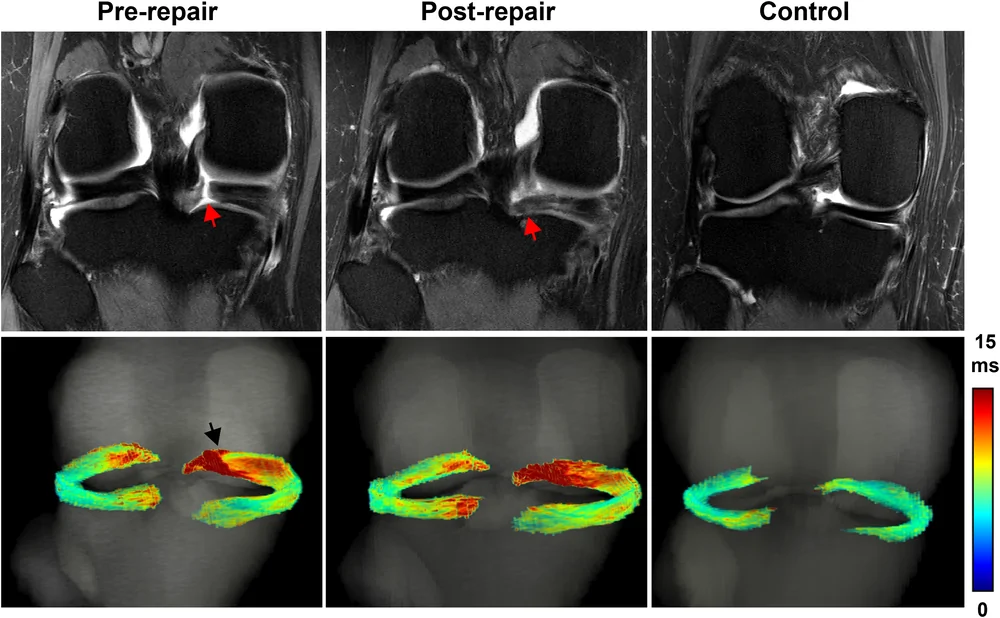

## 1 Introduction

It is now well recognized that nonoperative treatment of a medial meniscus posterior root tear (MMPRT) leads to a more rapid development of osteoarthritis and a higher risk for a total knee arthroplasty [1, 2]. When an MMPRT occurs, there is a loss of hoop stress, outward extrusion, and increased tibiofemoral contact pressures, ultimately accelerating osteoarthritis progression [3, 4, 5]. MMPRTs account for approximately 20% of all meniscal tears and often are caused by high degrees of knee flexion under increased tibiofemoral loading [6].

Although MMPRT repair has been reported to restore in situ joint mechanics and improve clinical outcomes, many postrepair patients exhibit increased medial meniscus extrusion [7, 8, 9], and those with increased meniscal extrusion exhibit a significant progression of radiographic osteoarthritis compared with those with no post‐repair extrusion [7]. Thus, identifying factors that lead to increased post‐repair meniscal extrusion is critical for developing treatments or techniques to optimize MMPRT repair.

To better understand the factors contributing to post‐repair extrusion, imaging methods capable of evaluating the intrinsic quality of meniscal tissue beyond macroscopic morphology are needed. Quantitative MRI, specifically T2* relaxation time mapping, is sensitive to collagen fiber organization and can provide insight into meniscal tissue integrity [10, 11, 12, 13]. Capturing these subtle T2 * differences across the meniscal subregions demands high spatial resolution and signal‐to‐noise ratio (SNR). Multiple studies have demonstrated two‐ to threefold higher SNR at 7 T compared with 3 T, with SNR scaling linearly to supra‐linearly with magnetic field strength [14, 15]. This improved SNR at clinical 7 T enables higher resolution assessment of meniscal microstructure.

Hager et al. [11] demonstrated using 7 T MR microscopy with direct histological comparison that T2* maps closely correspond to the spatial distribution of collagen fiber density and orientation: highly organized circumferential fibers exhibit short T2* values due to restricted proton mobility from residual dipolar coupling, while disorganized or degenerated fibers yield substantially longer T2* values. In line with this, Bae et al. [12] demonstrated that UTE T2* was significantly elevated in degenerated and torn versus normal menisci and correlated specifically with the histopathology of collagen fiber organization. Einarsson et al. [13] further experimentally demonstrated that T2* increases systematically with total Pauli score and is associated with the collagen fiber organization sub‐score. Together, these histologically validated studies establish the scientific evidence of the association between T2* and collagen fiber organization [10, 11, 12, 13].

A recent pre‐repair MMPRT patient study [16] at 7 T demonstrated significantly elevated T2* values in both the medial and lateral menisci of MMPRT patients, reflecting intrasubstance degeneration and disruption of collagen integrity. The purpose of this study was to conduct 3D T2* mapping to assess meniscus in post‐repair MMPRT patients and to compare these results with both pre‐repair T2* values and those from age‐ and gender‐matched healthy controls using 7 T MRI. In this study, we demonstrate that (1) pre‐repair T2* values will correlate with post‐repair medial meniscal extrusion; and (2) persistently elevated T2* values will be found in MMPRT patients both pre‐ and post‐repair compared with healthy controls, indicating that repair does not normalize T2* at early follow‐up.

## 2 Materials and Methods

### 2.1 Participants

In this IRB‐ and HIPAA‐compliant prospective study, informed consent was obtained from all participants who were enrolled consecutively between May 2021 and October 2024. All imaging and analysis endpoints for the present study were defined under a single IRB‐approved prospective protocol.

Patients were identified consecutively at the time of clinical evaluation in Twin Cities Orthopedics when diagnosed with a medial meniscus posterior root tear by an experienced orthopedic surgeon. All consecutive eligible patients who met inclusion criteria and provided written informed consent were enrolled.

Participants were eligible if they were ≥ 18 years old and underwent both a pre‐repair MRI (conducted prior to arthroscopic MMPRT repair) and a follow‐up MRI after the root repair. All patients had complete radial (type II) MMPRTs confirmed by MRI and arthroscopy [17]. The etiology was predominantly degenerative, consistent with the patient age distribution; a minority (*n* = 4) reported an acute inciting event. Healthy controls with no history of knee pathology or surgery were recruited to achieve a comparable age range (± 5 years) and sex distribution relative to the patient cohort (Figure 1). Exclusion criteria included pregnancy, contraindication to MRI (e.g., non‐MRI‐compatible implants), and any prior ipsilateral knee surgery.

**Figure 1:**
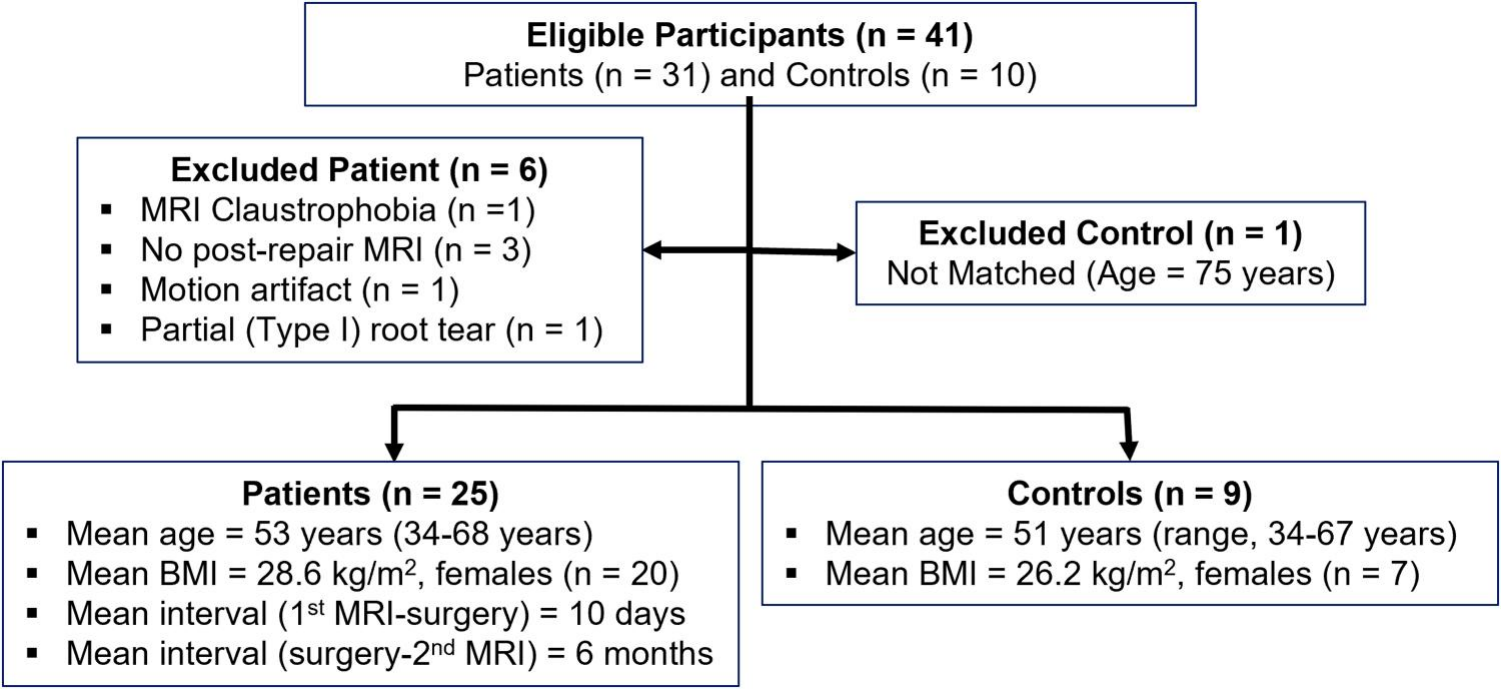
Flowchart of the study population.

### 2.2 MRI Protocol

MRI was performed pre‐ and post‐repair on a clinical FDA‐approved 7 T (Siemens, Erlangen, Germany) using a birdcage transmit/28‐channel receive phased‐array knee coil (Quality Electrodynamics, Mayfield Village, OH). The protocol consisted of standard clinical sequences and a 3D multi‐echo gradient‐recalled echo (GRE) sequence for quantitative 3D T2* mapping (Table 1). Healthy controls underwent a single MRI examination using the same imaging protocol. Moreover, the patients were positioned feet‐first in the supine position so that the main magnetic field (B0) was approximately perpendicular to the superior–inferior surfaces of the menisci, thereby minimizing the magic angle effect across meniscal tissue.

**Table 1:** List of MRI acquisition parameters.

Acquisition parameter	Coronal T_2_w FS TSE	Sagittal PDw TSE	Axial T_2_wFS TSE	Sagittal T2wFS Space	3D T2* mapping
Repetition time (ms)	5750	3000	6000	1500	26
Echo time (ms)	42	32	42	58	3.1, 6.1, 9.2, 12.2, 15.3, 18.4, 21.4
Flip angle (degrees)	140	130	180	140	15
Pixel bandwidth (Hz/pix)	265	560	320	765	360
Field of view (mm^2^)	139 × 139	139 × 139	129 × 129	125 × 125	130 × 130
Matrix size	496 × 372	496 × 347	384 × 307	272 × 272	300 × 300
In‐plane resolution (mm^2^)	0.14 × 0.14	0.14 × 0.14	0.17 × 0.17	0.46 × 0.46	0.44 × 0.44
Slice thickness (mm)	2.5	2.5	3	0.46	1.0
Slice gap (mm)	0.95	0.95	0.45	n/a	n/a
Number of slices	31	31	32	240	120
Echo train length	8	8	8	38	
GRAPPA/*CAIPIRINHA factor	2	3	2	*2 × 2	3
Number of averages	1	1	1	1	1
Imaging time (min:s)	3:55	1:18	2:36	8:57	5:18

Abbreviations: T_2_w FS TSE, T2‐weighted fat‐suppressed turbo‐spin echo; PDw, Proton‐density‐weighted; GRAPPA/CAIPIRINHA, GeneRalized autocalibrating partial parallel acquisition/controlled aliasing in parallel imaging results in higher acceleration.

### 2.3 Knee Examination, Arthroscopy, and Surgical Repair

All enrolled patients received a comprehensive knee evaluation by an experienced orthopedic surgeon (R.F.L.). In addition, patients were asked to complete the Knee Injury & Osteoarthritis Outcome Score (KOOS) [18] survey to report knee pain pre‐repair and again 6–12 months post‐repair.

Arthroscopic examination was performed during MMPRT repair to assess meniscal surface integrity and determine the extent of the tear preoperatively (Figure 2). Using the Pauli et al. [19] scoring system, the femoral and tibial surfaces and inner rims of both medial and lateral menisci were graded on a 0–3 scale: grade 0, smooth surface; grade 1, mild fibrillation; grade 2, moderate fibrillation; and grade 3, severe. All patients underwent a two‐tunnel transtibial root repair, with or without a centralization suture, as described previously [17]. To briefly describe here: two transtibial tunnels were drilled (Figure 2) (~4 cm distal to the joint line and 2 cm medial to the tibial tubercle) with cannulas into the decorticated medial meniscal root footprint. Self‐capture suture tapes were placed in a vertical mattress configuration at the edges of the root tear meniscal tissue and shuttled down the cannulas. The suture tapes were then tied over a cortical button on the anteromedial tibia. For patients with a centralization suture, a third transtibial tunnel was created [17] (~3 cm distal to the joint line and 3 cm medial to the tibial tubercle, exiting posteromedially near the medial tibial plateau articular margin), and one vertical mattress centralization suture was placed in the posteromedial meniscus, passed down the cannula in the tunnel, and fixed over a second cortical button.

**Figure 2:**
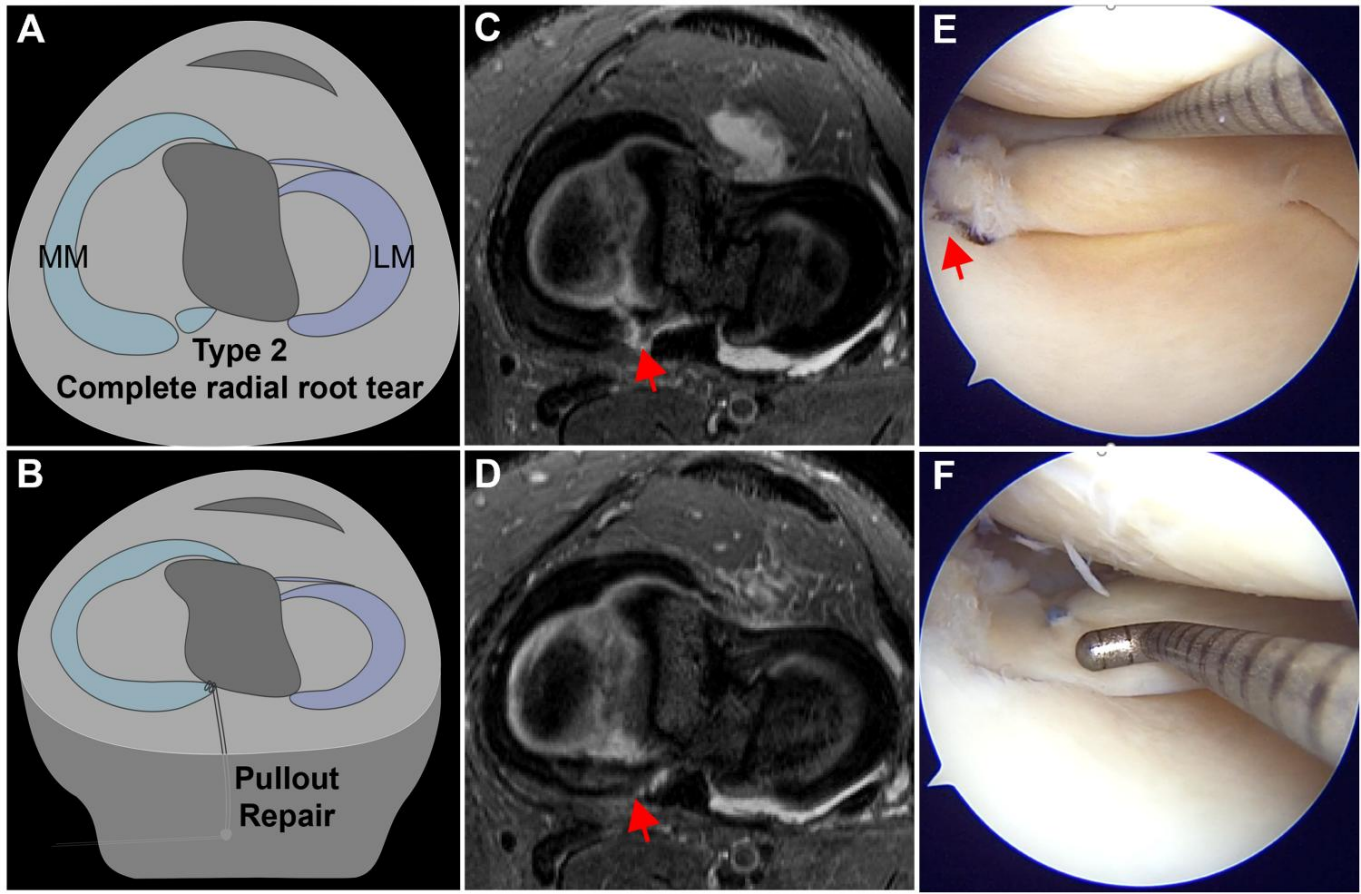
Representative schematics, MRI and arthroscopy images showing pre‐ and post‐repair of a medial meniscus posterior root tear (MMPRT) in a patient. (A) Axial schematic showing the MMPRT pre‐repair, and (B) its surgical attachment post‐repair. (C) Axial 3D SPACE showing MMPRT (arrow) pre‐repair, and (D) corresponding 3D SPACE showing root attachment post‐repair. (E–F) Arthroscopy images showing MMPRT (arrow) pre‐repair and the stitch attaching the root post‐repair, respectively.

### 2.4 MRI Extrusion Measurement

Two board‐certified musculoskeletal radiologists (T. T. and J. E.) independently assessed meniscal extrusion on pre‐ and post‐repair coronal T2‐weighted fat‐suppressed TSE images. To standardize extrusion measurements, all participants rested in a non‐weight‐bearing supine position for at least 30 min before MRI. Extrusion was consistently measured (in mm) between two parallel lines: one drawn along the peripheral margins of the medial tibial plateau (excluding osteophytes) and the other at the most peripheral edge of the meniscus (Figure 3A) on the coronal slice capturing the peak of the medial tibial eminence. The approach followed the method used in the Multicenter Osteoarthritis Study [20]. Both readers were blinded to the T2* mapping results.

**Figure 3:**
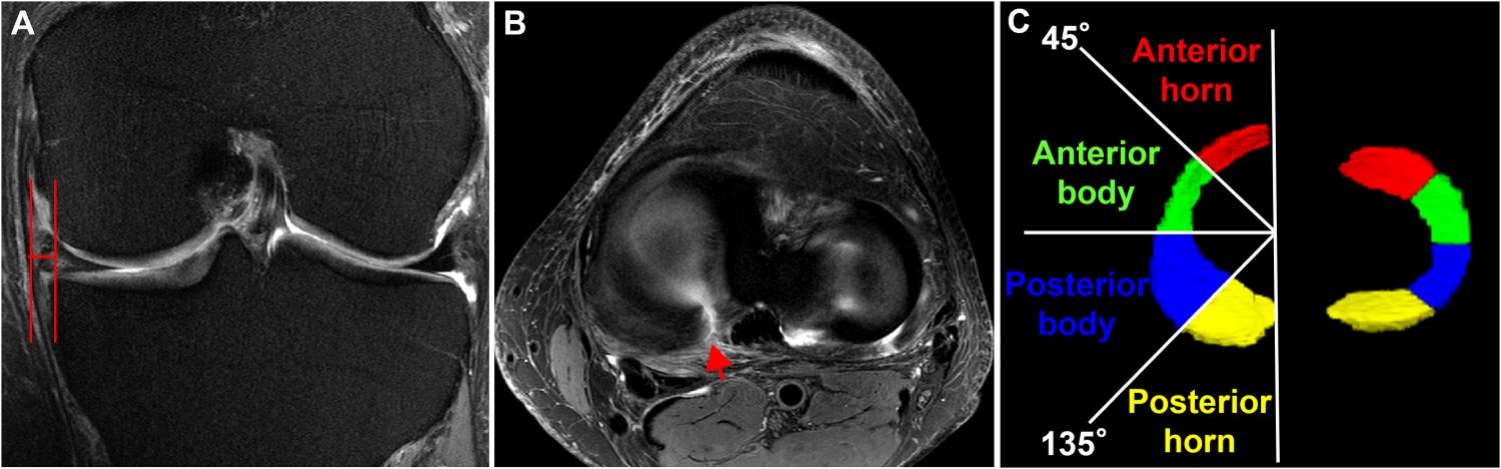
(A) Coronal T2 fat‐suppressed TSE image showing extrusion measurement between two vertical lines (in red). (B) Axial T2 fat‐suppressed TSE image showing MMPRT (arrow). (C) 3D regional reconstructed mask for quantitative T2* analysis of the medial and lateral menisci.

### 2.5 3D Segmentations and Quantitative T2* Mapping

3D segmentation of the medial and lateral menisci was performed using ITK‐SNAP [21] on the first echo time (TE = 3.1 ms) of T2*‐weighted images in three orthogonal planes, with 3D SPACE images serving as anatomic references to accurately delineate meniscal boundaries. In MMPRT patients, segmentation extended from the anterior horn (beginning about 1 mm proximal to the anterior root attachment) to the posterior region about one pixel (0.44 mm) proximal to the posterior root tear. In post‐repair images, an identical approach was used, but all voxels containing suture material were carefully identified as focal hypointense signal voids on early TE images and confirmed by progressive signal loss across all seven echoes times and were excluded prior to T2* fitting. Similarly, control menisci were segmented from 1 mm proximal to both anterior and posterior root attachment to closely match anatomical landmarks in the patients. Each meniscus was then sub‐divided into four angular regions relative to the line connecting the meniscal horn attachment points, as evaluated on the axial plane of the 3D projection of the segmentation: (i) anterior horn (0°−45°), (ii) anterior body (45°−90°), (iii) posterior body (90°−135°), and (iv) posterior horn (135°−180°) (Figure 3B,C).

Quantitative T2*‐maps were generated in MATLAB (MathWorks) by fitting the multi‐echo GRE signal to a mono‐exponential decay model using non‐linear least squares optimization. Reproducibility of T2* mapping has been extensively evaluated using scan–rescan measurements and reported previously [16]. Fitting accuracy was evaluated by the root‐mean‐square error (RMSE) and meniscal volumes were derived from the voxel counts.

### 2.6 Statistical Analysis

The sample size of this study was determined by a prospective power analysis of the parent randomized controlled trial [17], which established that 24 patients were required to detect a significant difference in postoperative medial meniscal extrusion between transtibial root repair with and without a centralization suture. The quantitative T2* mapping analysis reported here was a pre‐planned secondary analysis conducted within the same prospectively powered, IRB‐approved study and imaging protocol.

Comparisons were performed using non‐parametric Kruskal–Wallis tests for meniscus volume and the T2* values between meniscal regions in patients pre‐ and post‐repair, and corresponding regions in the controls. The *p*‐values were adjusted for pairwise comparisons using Dunn–Bonferroni correction. As extrusion data followed a normal distribution, confirmed by both Kolmogorov–Smirnov and Shapiro–Wilk tests, a paired *t*‐test was used to compare pre‐repair against post‐repair values. Spearman rank correlation (ρ) was used to evaluate the correlation analysis. Inter‐reader agreement for meniscal extrusion measurements performed by two radiologists was assessed using the intraclass correlation coefficient (ICC) and Bland–Altman analysis. A *p*‐value < 0.05 was considered statistically significant. All statistical analyses were performed using SPSS (version 28, IBM Corp, Armonk, NY).

## 3 Results

### 3.1 Participant Characteristics

Of 31 patients initially enrolled, one was excluded due to claustrophobia, three did not return for follow‐up post‐repair MRI, one had partial (type I) root tear [22], and one had motion artifacts (Figure 1). The remaining 25 patients (mean age, 53 years ± 8 years; mean BMI, 28.6 kg/m^2^; 20 female) were included in the analysis and underwent 7 T MRI before repair (mean, 10 days ± 11 days) and post‐repair. Post‐repair MRI was performed at a mean of 6 months ±2 months (range, 4–10 months). Additionally, of 10 initially enrolled controls, one was excluded due to age mismatch, and nine controls (mean age, 51 years ± 11 years; mean BMI, 26.2 kg/m^2^, seven females) were included (Figure 1).

All included pre‐repair patients had complete radial MMPRTs (type II root tears [22]) confirmed by both MRI and arthroscopy. Two board‐certified, musculoskeletal fellowship–trained radiologists independently reviewed all cases. Both agreed that all repairs met imaging criteria for integrity: intact suture fibers at the tibial anchor with no fluid at the repair interface [23].

### 3.2 MRI Extrusion Assessment

The intraclass correlation coefficient between the two meniscal extrusion readers was 0.958 (95% CI: 0.925, 0.976), indicating excellent inter‐reader agreement. The consistency in meniscal measurement between the two readers is depicted with Bland–Altman plots (Figure 4). Medial extrusion significantly increased from 3.7 ± 1.1 mm pre‐repair to 4.9 ± 1.5 mm post‐repair, corresponding to a mean increase of +1.2 mm (95% CI: 0.49, 1.91 mm) (*p* = 0.002). This increase in extrusion was observed across patients despite MRI‐intact root repairs.

**Figure 4:**
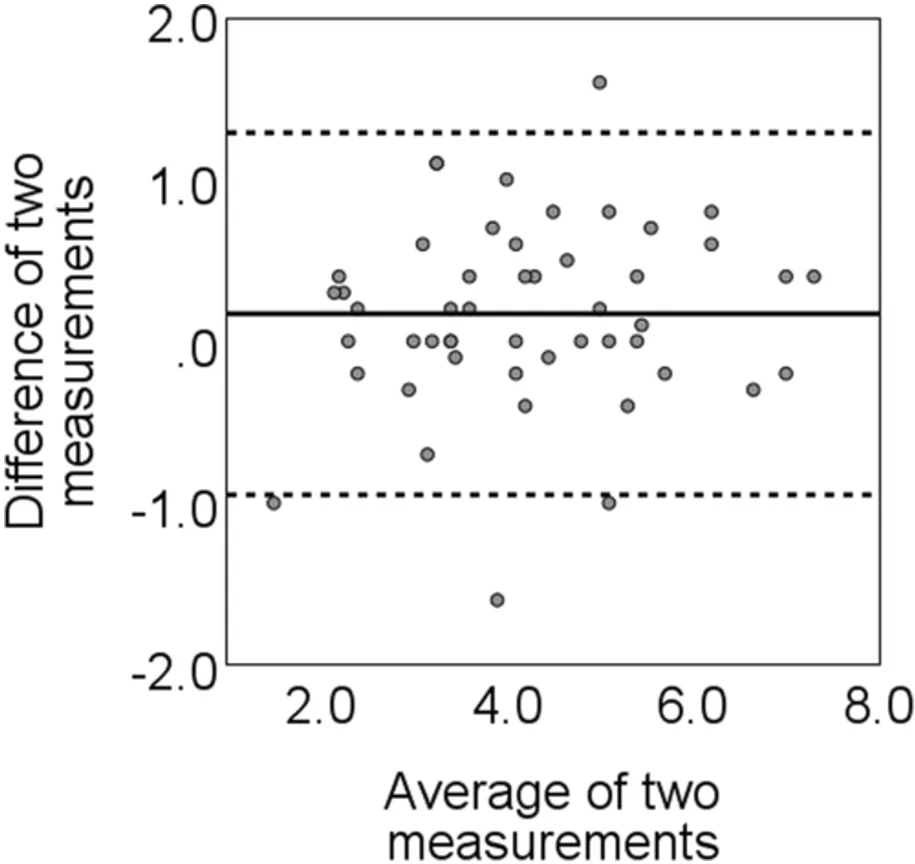
Bland–Altman plot of the meniscal extrusion measurements by two musculoskeletal radiologists. The solid line is the mean difference (d), and the dotted lines are d − 1.96 SD (lower) and d + 1.96 SD (upper). (SD = standard deviation).

Since meniscal extrusion was measured at the level of the medial meniscus body, the mean of the median T2* values from the anterior body and posterior body subregions were used for correlation analysis. Post‐repair meniscal extrusion measurements were strongly and positively correlated with both pre‐repair (ρ = 0.765; 95% CI: 0.520, 0.893; *p* < 0.001) and post‐repair (ρ = 0.698; 95% CI: 0.408, 0.860; *p* < 0.001; respectively) T2* values (Figure 5). None of the healthy controls exhibited medial or lateral meniscal extrusion. Lateral meniscal extrusion was not a prespecified outcome as it was not a recognized consequence of MMPRT. The effect of the centralization suture on post‐repair extrusion is reported in a separate randomized controlled trial study [17].

**Figure 5:**
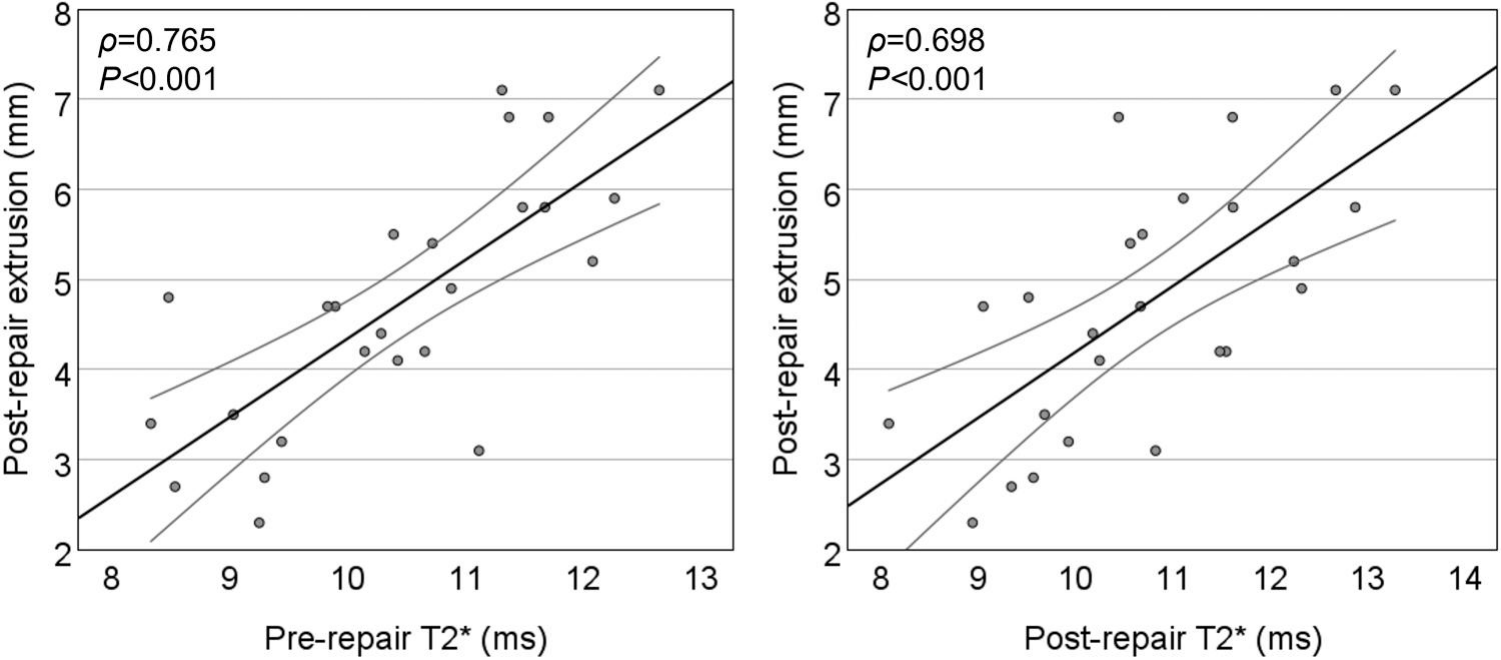
Scatter plot shows the correlation of post‐repair meniscal extrusion with pre‐ and post‐repair T2* values. The solid lines represent linear regression fits with 95% confidence intervals (light gray).

### 3.3 Quantitative Meniscal T2* Evaluation

Significantly higher T2* values were observed across medial and lateral menisci in both pre‐ and post‐repair patients when compared with the controls ([medial meniscus: anterior body *p* < 0.05; anterior horn, posterior body and posterior horn all *p* < 0.01]; [lateral meniscus: anterior body and posterior body *p* < 0.05; anterior horn and posterior horn *p* < 0.01]) (Figure 6). No significant differences in T2* values were noted between pre‐ and post‐repair patients.

**Figure 6:**
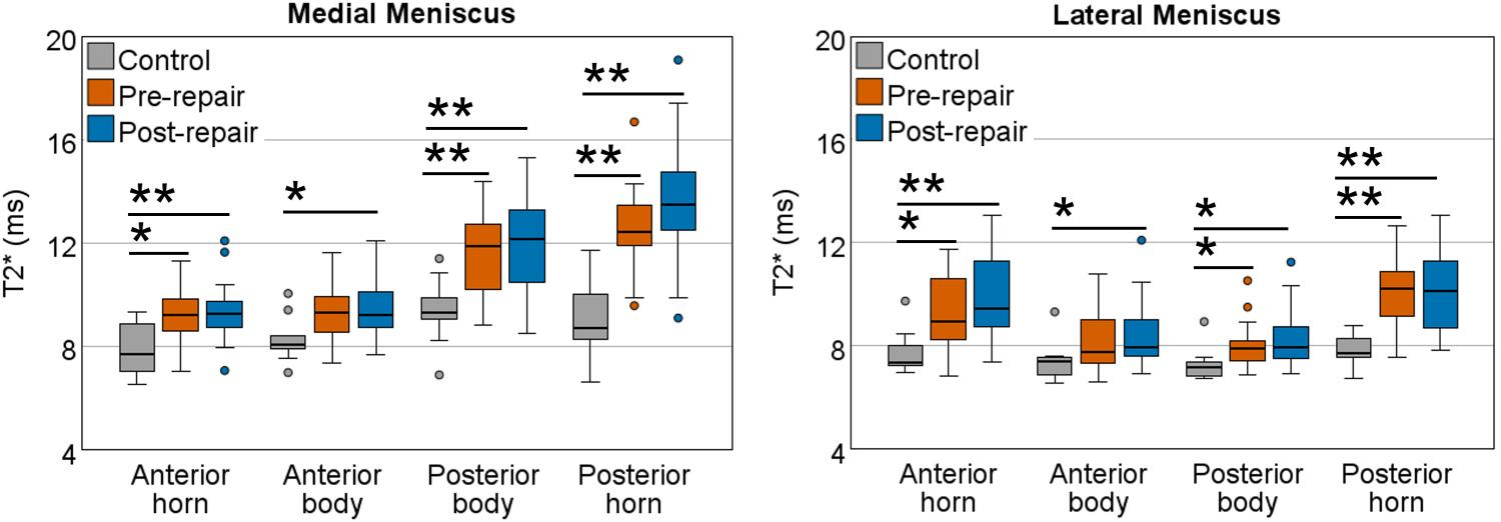
Boxplots of the T2* values comparing analyzed meniscal regions of patients with medial meniscus posterior root tears (MMPRTs) pre‐repair and post‐repair, as well as healthy controls. The T2* relaxation times in patients both pre‐ and post‐repair were significantly longer (*p < 0.05; **p < 0.001) when compared to the controls across both menisci.

The largest regional differences between patients and controls were observed in the posterior body and posterior horn of the medial meniscus. Figure 7 presents 3D visualization of these regional differences.

**Figure 7:**
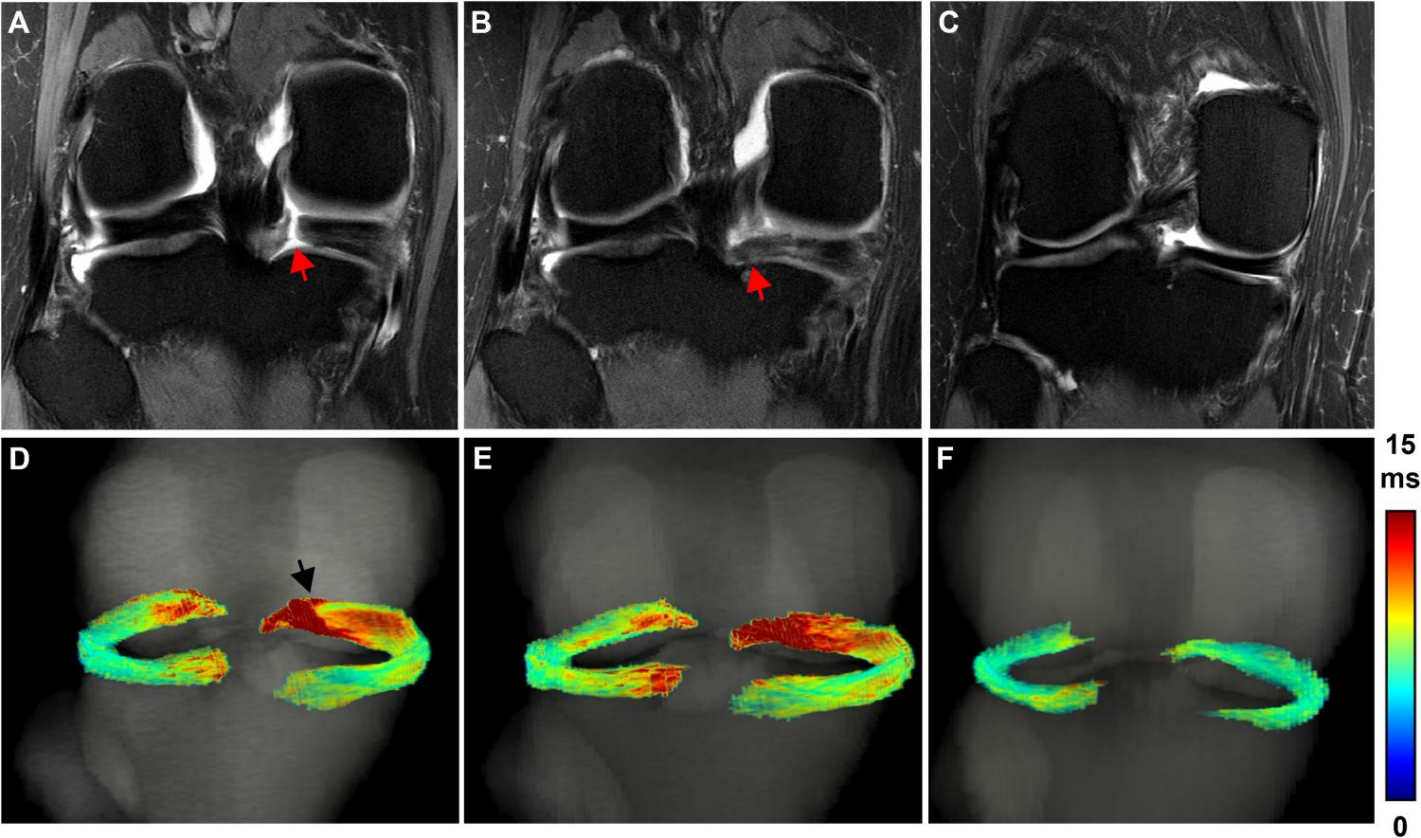
Representative coronal T2 fat‐suppressed TSE images (A–C) and 3D visualizations of T2* maps (D‐F) from (A, D) a pre‐repair patient, (B, E) corresponding post‐repair patient, and (C, F) a healthy control overlaid on segmented, coronal semi‐transparent femorotibial bone structures. The T2* maps highlight the elevated T2* values adjacent to the root tear (arrows) region both before and after repair when compared with the control.

No significant difference in T2* was observed between patients with (*n* = 14, mean T2* ± SD = 10.9 ± 1.5) and without (*n* = 11, 11.3 ± 0.8) a centralization suture [17].

Both pre‐ and post‐repair patients had significantly larger medial (*p* = 0.001, *p* = 0.008, respectively) and lateral meniscal volumes (*p* = 0.001, *p* = 0.001) compared with the controls, respectively (Table 2). While volumes were significantly higher in patients with MMPRTs compared with the controls, no significant differences in volumes were noted between pre‐ and post‐repair patients.

**Table 2:** Comparison of mean ± standard deviation (SD) of meniscal volumes across participants.

Regions		Patients		Controls
		Pre‐repair	Post‐repair	
Medial Meniscus	Pixel count	48963 ± 12419	46543 ± 14367	29946 ± 7881
	cm3	2.37 ± 0.60	2.25 ± 0.70	1.45 ± 0.38
Lateral Meniscus	Pixel count	37106 ± 12349	36576 ± 12585	20309 ± 5728
	cm3	1.80 ± 0.60	1.77 ± 0.61	0.98 ± 0.28

The average normalized RMSE for the mono‐exponential T2* fitting across all evaluated meniscal sub‐regions and all participants was 3.8% ± 0.7% (range: 2.4%–6.4%), indicating reliable model fitting. Additionally, a previous study [16] reported good to excellent reproducibility of 7 T T2* measurements in the meniscal regions, with scan‐rescan ICC values of 0.87–0.97.

### 3.4 Intraoperative Arthroscopic Evaluation

Arthroscopic examination revealed Pauli grades 1–2 on the medial meniscal surface in four patients (all patients with grade 1), while no visible abnormalities were observed in the remaining 21 patients.

No significant correlation (*ρ* = 0.372; 95% CI: –0.039, 0.676; *p* = 0.067) was found between pre‐repair T2* values and Pauli grades.

### 3.5 Patient Reported Outcome

Of the 25 patients, 19 completed the KOOS pain survey both pre‐repair and 6–12 months post‐repair. There was no significant difference (*p* = 0.805) between the change in KOOS pain scores from the pre‐repair to 6‐month post‐repair and from pre‐repair to 12‐month post‐repair scores.

No significant correlation was found between pre‐repair or post‐repair medial meniscus mean T2* values and the change from pre‐repair to post‐repair KOOS pain scores (*ρ* = –0.060, 95% CI: –0.511, 0.418, *p* = 0.808; ρ = –0.170, 95% CI: –0.589, 0.320, *p* = 0.488; respectively).

## 4 Discussion

The primary finding of this study was that pre‐repair T2* values were strongly correlated with post‐repair medial meniscal extrusion, demonstrating that baseline meniscal tissue quality is associated with post‐repair extrusion. Although the volumetric differences were statistically significant between patients and the controls, their absolute magnitude and clinical significance are uncertain, likely reflecting a combination of edema and alterations associated with the root tear and its repair. In parallel, meniscal T2* values remained significantly elevated compared with healthy controls at the 6‐month post‐repair time point and showed no significant change from pre‐repair T2* values, indicating that T2* values had not normalized at this early time point, consistent with persistent matrix alterations. Experimental and clinical studies [24, 25, 26] have demonstrated that it takes longer than 6 months for meniscal T2* values to normalize after repair, particularly in the medial posterior horn, where persistent elevation may reflect slower or incomplete tissue recovery. The study by Fedje‐Johnston [24] further suggests that T2* normalization is associated with histologically confirmed improvements in collagen organization and proteoglycan content.

A fundamental and unresolved question in the field is whether meniscal extrusion causes posterior root tears or whether root tears cause extrusion. Several converging lines of evidence support a pre‐existing generalized degenerative process rather than a purely post‐tear phenomenon. In the current study, elevated T2* values were observed not only in the involved medial meniscus but also in the structurally intact lateral meniscus, suggesting whole‐joint matrix degeneration rather than localized injury alone, which is also reported in a previous meniscus T2* study at 7 T [16]. Arno et al. [27] similarly demonstrated strong associations between meniscal T2* and osteoarthritis severity, extrusion, and reduced tibiofemoral contact area. Chronologically, Krych et al. [28] showed that meniscotibial ligament disruption and extrusion preceded posterior root tear identification on serial MRI by a mean of 1.7 years, supporting the concept that root failure represents the endpoint of an evolving degenerative cascade. This interpretation is indirectly reinforced by Doan et al. [29], who demonstrated in a cadaveric model using otherwise intact menisci that root repair alone substantially restored native contact mechanics after experimentally created root injury. Because that model lacked chronic intrameniscal degeneration before transection, the favorable biomechanical restoration observed experimentally contrasts with the persistent extrusion and elevated T2* values observed clinically in the present study, suggesting that pre‐existing matrix degeneration contributes substantially to postoperative biomechanical failure in degenerative MMPRT patients.

The paradoxical increase in extrusion despite MRI‐intact repair is best explained by a biology‐limited mechanism. As demonstrated in a prior study by Kajabi et al. [16], T2* elevation extends far beyond the immediate root tear site, indicating widespread collagen network disorganization that makes the meniscal tissue vulnerable to extrusion independent of the repair status. Restoring the root attachment may normalize the anatomical boundary condition but cannot immediately reconstitute the collagen fiber organization required for effective hoop stress resistance and meniscal containment under physiological loading.

Such imaging‐based characterization may ultimately inform patient stratification for adjunctive procedures, including centralization sutures or biologic augmentation [17, 30] in addition to a standard transtibial root repair [31]. No significant association was observed between pre‐repair meniscal T2* values and post‐repair pain improvement. This finding indicates that elevated T2* values reflect tissue‐level microstructural alterations rather than short‐term patient‐reported symptoms, providing structural information that is complementary to clinical outcome measures.

Meniscal repair in general also carries a meaningful failure/retear risk with pooled failure rates of approximately 20%–25% reported at ≥ 5 years, but substantially higher rates—up to ~48%—described in certain cohorts and earlier all‐inside repair techniques [32, 33, 34, 35, 36], consistent with a biology‐limited explanation for post‐repair extrusion. It should be noted that MMPRTs are predominantly degenerative and typically occur in middle‐aged individuals with underlying joint degeneration [16]. The T2* findings in this study may not generalize to traumatic meniscal tears (e.g., bucket‐handle tears) in younger athletes where baseline tissue quality is likely to be substantially higher.

Meniscal root repair lowers the risks of OA and total knee arthroplasty (TKA) compared with meniscectomy or nonoperative care, yet the absolute risk for the progression of OA after repair remains substantial (~53% OA, 33.5% TKA at 10 years) [1]. Long‐term natural‐history data for nonoperative and degenerative MMPRTs demonstrate poor outcomes (~95% OA at ≥ 10 years; 64% conversion to TKA) [2]. Another study [37] reported “intact‐but‐lax” phenomena in 41% of patients on second‐look arthroscopy 1 year after surgery. Notably, patients with intact but lax repairs showed an average medial joint space narrowing of 0.7 mm, indicating that residual laxity and meniscal extrusion lead to compromised cartilage integrity [37]. Consistent with these findings, the present study found prolonged T2* values in the menisci at a mean post‐repair follow‐up of 6 months, indicating persistent collagen fiber disorganization.

The linkage between extrusion and osteoarthritis progression is well established and increasingly central to clinical decision‐making [4, 5, 20, 38]. In vivo series reported that extrusion commonly persists after a medial meniscus root repair [7, 8, 31]. This argues for a potential tissue‐quality‐first evaluation of MMPRTs using quantitative T2* mapping to triage which patients might benefit from centralization sutures [17] or other surgical augmentation [30, 31, 38]. Notably, pre‐surgical reports in this study documented only mild to moderate medial meniscal surface fibrillation at the time of surgery, underscoring that surface inspection underestimates subsurface matrix disorganization detectable with quantitative MRI [16].

Ultrahigh field 7 T MRI is not yet universally available; and cost, patient access, and implant compatibility represent real practical constraints at this time. This study notes, however, that advances in quantitative MRI have historically followed a pattern in which knowledge generated at the technological frontier, where signal fidelity and spatial resolution are sufficient to resolve the biology, subsequently informs what is achievable and meaningful at clinical field strengths. The submillimeter resolution and SNR afforded by 7 T in about 5 min acquisition are what make whole‐meniscus 3D T2* mapping of thin subregions feasible within a clinically acceptable scan time, and the histological literature establishing the biological meaning of T2* in meniscal tissue was itself generated at high and ultrahigh field strengths [10, 11, 13]. While arthroscopy continues to be the gold standard for the evaluation of MMPRTs, advances in 7 T MRI are making diagnosis increasingly time efficient, sensitive, and specific [39, 40]. The findings reported here define imaging biomarkers that can now be pursued with optimized protocols at 3 T, where the clinical reach is far broader. The number of clinical 7 T installations has grown substantially since FDA clearance and is projected to continue expanding at tertiary centers.

This study has some limitations. First, the ~6‐month interval represents an early remodeling window and longer follow‐up [24, 25, 26] is required to define recovery trajectories and clinical thresholds. Additionally, the time between symptom onset or injury and the pre‐repair MRI was not systematically recorded, which prevented analysis of symptom duration as a covariate. Second, histologic or biomechanical reference standards to in vivo MRI findings are lacking; therefore, biological interpretation is based on prior rigorous ex vivo correlations between relaxation times and collagen fiber orientation [10, 11, 12, 41, 42]. Age and gender matching was applied at the group level rather than through formal covariate adjustment; residual confounding by these variables cannot be entirely excluded. Finally, T2* is sensitive to orientation (magic‐angle effects) and B0 inhomogeneity, which were mitigated through standardized positioning, whole‐meniscus 3D sampling, and B0 shimming, which was performed in all participants with inspection of B0 maps.

In conclusion, high‐resolution 3D T2* mapping at 7 T revealed persistently elevated T2* values after MMPRT repair, consistent with ongoing collagen fiber disorganization based on ex‐vivo literature correlating T2* with collagen fiber orientation. Pre‐repair T2* values were strongly correlated with early post‐repair meniscal extrusion despite MRI‐intact repairs, indicating that preoperative tissue quality is associated with postoperative outcomes. These findings support quantitative T2* mapping as a prognostic imaging biomarker that complements conventional morphologic MRI in the evaluation of MMPRTs.

## Author Contributions

**Abdul Wahed Kajabi:** substantial contributions to research design, acquisition, analysis and interpretation of data, drafting the paper and revising it critically, and approval of the submitted and final versions. **Karsten Knutsen:** substantial contributions to analysis and interpretation of data, revising the paper critically, and approval of the submitted and final versions. **Eisa Hedayati:** substantial contributions to analysis and interpretation of data, revising the paper critically, and approval of the submitted and final versions. **Collin Steinberger:** substantial contributions to analysis and interpretation of data, revising the paper critically, and approval of the submitted and final versions. **Štefan Zbýň:** substantial contributions to analysis and interpretation of data, revising the paper critically, and approval of the submitted and final versions. **Asif Abul Hassan:** substantial contributions to analysis and interpretation of data, revising the paper critically, and approval of the submitted and final versions. **Hassan Ahad:** substantial contributions to analysis and interpretation of data, revising the paper critically, and approval of the submitted and final versions. **Abhinav Lamba:** substantial contributions to analysis and interpretation of data, revising the paper critically, and approval of the submitted and final versions. **Luke V. Tollefson:** substantial contributions to analysis and interpretation of data, revising the paper critically, and approval of the submitted and final versions. **Takashi Takahashi:** substantial contributions to analysis and interpretation of data, revising the paper critically, and approval of the submitted and final versions. **Gregor J. Metzger:** substantial contributions to research design, acquisition, analysis and interpretation of data, revising the paper critically, and approval of the submitted and final versions. **Robert F. LaPrade:** substantial contributions to research design, acquisition, analysis and interpretation of data, revising the paper critically, and approval of the submitted and final versions. **Jutta M. Ellermann:** substantial contributions to research design, acquisition, analysis and interpretation of data, revising the paper critically, and approval of the submitted and final versions. All authors have read and approved the final submitted manuscript.

## Data Availability

The data that support the findings of this study are available on request from the corresponding author. The data are not publicly available due to privacy or ethical restrictions.
